# Associations of Fish Oil Supplement Use With Testicular Function in Young Men

**DOI:** 10.1001/jamanetworkopen.2019.19462

**Published:** 2020-01-17

**Authors:** Tina Kold Jensen, Lærke Priskorn, Stine A. Holmboe, Feiby L. Nassan, Anna-Maria Andersson, Christine Dalgård, Jørgen Holm Petersen, Jorge E. Chavarro, Niels Jørgensen

**Affiliations:** 1 Department of Environmental Medicine, University of Southern Denmark, Odense, Denmark; 2 Department of Growth and Reproduction, Rigshospitalet, University of Copenhagen, Copenhagen, Denmark; 3 International Center for Research and Research Training in Endocrine Disruption of Male Reproduction and Child Health, Rigshospitalet, University of Copenhagen, Copenhagen, Denmark; 4 Department of Nutrition, Harvard T.H. Chan School of Public Health, Boston, Massachusetts; 5 Department of Environmental Health, Harvard T.H. Chan School of Public Health, Boston, Massachusetts; 6 Section of Biostatistics, Department of Public Health, University of Copenhagen, Copenhagen, Denmark; 7 Department of Epidemiology, Harvard T.H. Chan School of Public Health, Boston, Massachusetts; 8 Channing Division of Network Medicine, Brigham and Women’s Hospital and Harvard Medical School, Boston, Massachusetts

## Abstract

**Importance:**

Many young men have poor semen quality, and the causes are often unknown. Supplement intake of ω-3 polyunsaturated fatty acid has been found to improve semen quality among men with infertility, but the association with semen quality among healthy men is unknown.

**Objective:**

To determine if intake of ω-3 fatty acid supplements is associated with testicular function as measured by semen quality and reproductive hormone levels among healthy men.

**Design, Setting, and Participants:**

This cross-sectional study included young Danish men from the general population recruited between January 1, 2012, and December 31, 2017, at compulsory examinations to determine their fitness for military service. Young unselected men were approached after the examination and invited to participate in a study of reproductive function, regardless of their fitness for military service. Data analysis was conducted from September 1, 2018, to June 30, 2019.

**Exposures:**

Intake of supplements, including fish oil, during the past 3 months.

**Main Outcomes and Measures:**

Semen quality, measured as volume, concentration, total sperm count, percentage of morphologically normal spermatozoa, and motility, and serum reproductive hormone levels, measured as follicle-stimulating hormone, luteinizing hormone, testosterone, free testosterone, and inhibin B levels.

**Results:**

Among 1679 young Danish men (median [interquartile range] age, 18.9 [18.7-19.4] years) recruited to participate, 98 men (5.8%) reported use of fish oil supplements during the past 3 months, of whom 53 (54.1%) reported intake on 60 or more days. After adjustment and compared with men with no supplement intake, men with fish oil supplement intake on fewer than 60 days had semen volume that was 0.38 (95% CI, −0.03 to 0.80) mL higher, and men with fish oil supplement intake on 60 or more days had semen volume that was 0.64 (95% CI, 0.15 to 1.12) mL higher (*P* for trend < .001). Similarly, testicular size in men with supplement intake on fewer than 60 days was 0.8 (95% CI, −0.2 to 1.9) mL larger and in men with fish oil supplement intake on 60 or more days was 1.5 (95% CI, 0.2 to 2.8) mL larger compared with men with no supplement intake (*P* for trend = .007). After adjustment, men with fish oil supplement intake had a 20% (95% CI, 9%-31%) lower follicle-stimulating hormone level and 16% (95% CI, 8%-24%) lower luteinizing hormone level compared with men with no supplement intake. There were no associations of intake of other supplements with measures of testicular function.

**Conclusions and Relevance:**

These findings suggest that intake of fish oil supplements was associated with better testicular function, which is less likely to be due to confounding by indication, as no associations of intake of other supplements with testicular function were found. This cross-sectional study did not examine the actual content of ω-3 fatty acids in the supplements; therefore, these findings need confirmation in well-designed randomized clinical trials among unselected men.

## Key Points

**Question:**

Is intake of fish oil supplements associated with testicular function as measured by semen quality and reproductive hormone levels among young healthy men?

**Findings:**

In this cross-sectional study including 1679 young men in Denmark, fish oil supplements were associated in a dose-response manner with higher semen volume and total sperm count, larger testicular size, a higher calculated free testosterone to luteinizing hormone ratio, and lower follicle-stimulating hormone and luteinizing hormone levels after adjusting for confounders.

**Meaning:**

These findings suggest that healthy men may benefit from intake of fish oil supplements, but a well-designed randomized clinical trial among unselected men is warranted.

## Teaser

This cross-sectional study examines the associations of fish oil supplement intake with testicular function as measured by semen quality and reproductive hormone levels in young men in Denmark.

## Introduction

Infertility affects approximately 15% of all couples, and approximately 40% to 50% of these fertility issues are due to male factors.^1,2^ Also, a decline in semen quality has been documented during the past 50 to 70 years^3,4^; however, large geographic and population differences exist. The causes for the decline in semen quality are likely multifactorial and may include lifestyle and behavioral changes as well as possible exposure to chemicals with endocrine disrupting abilities.^5,6,7^ A 2017 systematic review^8^ found that a healthy diet rich in certain nutrients, such as ω-3 fatty acids, antioxidants (eg, vitamin E, vitamin C, β-carotene, selenium, zinc, cryptoxanthin, and lycopene), other vitamins (eg, vitamin D and folate) and low in saturated fatty acids and trans–fatty acids was associated with good semen quality. Fish, shellfish, other seafood, poultry, cereals, vegetables, fruits, low-fat dairy, and skim milk have been found to be positively associated with several semen parameters.^9^ However, fish and fish liver oil in particular also contain other essential nutrients, including retinoic acid (ie, vitamin A), that have been associated with better semen quality.^10^ In addition, diets rich in processed meats, soy foods, potatoes, full-fat and total dairy products, cheese, coffee, alcohol, sugar-sweetened beverages, and sweets have been associated with worse semen quality.^8^ However, it is difficult to assess whether these associations are causal.

Two studies among men from couples experiencing infertility have addressed the association of supplementation with ω-3 polyunsaturated fatty acid (PUFA) on semen quality,^11,12^ and both reported higher sperm concentration, total sperm count, and motility. One of these studies^12^ also reported a higher number of morphologically normal spermatozoa. In 2 randomized clinical trials (RCTs),^12,13^ supplementation with PUFA docosahexaenoic acid (DHA) did not improve traditional semen quality measures but decreased sperm DNA fragmentation index and increased the number of progressively motile spermatozoa. In 2 parallel 2-group dietary intervention trials,^14,15^ healthy young men were randomized to daily intake of a mix of nuts or walnuts, which are rich in PUFA and monounsaturated fatty acids, vs usual diet for 12 to 14 weeks; both studies reported increased total sperm count and motility and higher proportions of morphologically normal spermatozoa in the intervention groups.

To our knowledge, no studies on the association of intake of ω-3 fatty acids supplements with semen quality among healthy men from the general population have been performed. Therefore, we examined the association of intake of fish oil supplements, which are high in ω-3 fatty acid, with testicular function as measured by semen quality and reproductive hormone levels.

## Methods

The study was conducted in accordance with the Declaration of Helsinki,^16^ and ethical approval was obtained from the local ethical committee for the region of Southern Denmark. All participants provided written informed consent.

Because of the military conscription system in Denmark, all men aged 18 years, except those experiencing severe chronic illness, are required to undergo a physical examination to determine their fitness for military service. From January 1, 2012, to December 31, 2017, we approached young men during this examination, regardless of their fitness for military service, and invited them to participate in a study of testicular function. Men who agreed to participate were given an appointment for examination at the Department of Growth and Reproduction at Rigshospitalet, Copenhagen, Denmark, and were compensated 500 kr (approximately US $74) for their time. Prior to the day of study participation, all participants completed a questionnaire. On the day of study participation, they delivered a semen sample, had a blood sample drawn, and underwent a physical examination. The study has been ongoing since 1996, but in 2012 the questionnaire was revised to include information on nutritional supplements.^17^ This study followed the Strengthening the Reporting of Observational Studies in Epidemiology (STROBE) reporting guideline.

### Questionnaire

All participants completed a questionnaire on health, lifestyle, and diet before the examination. This included information on previous or current health conditions and genital conditions, such as inguinal hernia, varicocele, epididymitis, and having undergone a surgical procedure to treat testicular torsion. They were asked if they had experienced gonorrhea or chlamydia and if they were born with both testicles in the scrotum. In addition, they reported whether they had experienced a fever of 38.0 °C or higher within the previous 3 months.

The men were asked about their frequency of smoking tobacco or marijuana (ie, daily, occasionally, or never) in the last 3 months and intake of alcoholic beverages during each day of the past week. Weekly alcohol intake was calculated as the sum of the reported intake across all beverages.^18^ Men were also asked to rate their general health and physical fitness (ie, very good, good, average, poor, or very poor). Finally, men were asked to query their mothers regarding whether their mothers had smoked during pregnancy.

The men stated whether they used vitamins or dietary supplements within the last 3 months. If so, the name of the product, the dose, and the reason for use were noted, as well as the number of days taking the supplement. Supplement intake was coded manually by one of us (T.K.J.) without prior knowledge of the testicular function of the young men. Answers were categorized into no supplement intake, multivitamins, fish oil supplements, vitamin C supplements, vitamin D supplements, or other supplement intake.

### Physical Examination

Testis size was measured by trained physicians using ultrasonography, and varicocele (grades 1-3) was assessed by palpation. Also, hydrocele and location of the testes in the scrotum were determined. Waist and hip circumference were measured, as were weight and height, from which waist-to-hip ratio and body mass index (BMI, calculated as weight in kilograms divided by height in meters squared) were calculated.

### Semen Analysis

All men provided a semen sample by masturbation in a room close to the semen laboratory. They had been asked to abstain from ejaculation for at least 48 hours before sample delivery. However, they were not excluded if this was not the case, and we accounted for the reported abstinence time. The semen sample was kept at 37 °C until analysis as described by Priskorn et al,^17^ which is in accordance with the most recent guideline from the World Health Organization,^19^ as previously described.^20^ In short, semen volume was assessed by weighing, sperm concentration was determined in diluted samples using a Bürker-Türk hemocytometer, and the total sperm count was calculated (semen volume × sperm concentration). For sperm motility, 2 drops of well-mixed semen were placed on a glass slide and examined under the microscope, and the spermatozoa were classified as progressively motile, locally motile, or immotile. Fixed and Papanicolaou-stained morphologic slides were prepared and evaluated according to strict criteria.^21^ For all assessments, measurements were performed twice in the same sample, and the mean was used.

### Reproductive Hormones

A fasting blood sample was taken at the day of examination in the morning, with a median (interquartile range [IQR]) sampling time of 10:20 (9:40-11:10) am. Serum concentrations of follicle-stimulating hormone (FSH), luteinizing hormone (LH), sex hormone-binding globulin, testosterone, and estradiol were determined using a time-resolved immunofluorometric assay (Delfia; Wallac). Starting in 2014, estradiol levels were assessed with a radioimmune assay (Pantex), and testosterone and sex hormone-binding globulin levels were assessed by chemiluminescent immunoassays (Access2; Beckman Coultier). Inhibin B levels were determined by a specific 2-sided enzyme-immunometric assay (Inhibin B gen II; Beckman Coulter). All hormone levels were analyzed in the same laboratory. The hormone levels were analyzed yearly in batches, including reanalysis of a number of controls from the previous year to ensure comparability over time and among the different assays. Free testosterone levels were calculated based on the measured serum concentrations of total testosterone and sex hormone-binding globulin and assuming a fixed albumin value according to Vermeulen,^22^ and the ratios of inhibin B to FSH and free testosterone to LH were calculated.

### Statistical Analysis

Initially, semen parameters, testis size, and reproductive hormone levels were compared among men stratified by intake of different supplements by medians and IQR, with dietary supplement use during the past 3 months categorized as no intake, multivitamins, fish oil supplements, vitamin C supplements, and vitamin D supplements, or other supplement intake. We subsequently compared men who used no supplements with men who used other supplements (including vitamin D, vitamin C, and multivitamins) because we expected supplement users to be healthier than men with no supplement use or fish oil supplement use. Furthermore, men were categorized according to whether they had used fish oil supplements for fewer than 60 days or 60 or more days during the past 3 months. We then compared the distributions of the variables from the questionnaires and physical examinations among men with use of different supplements (ie, none, fish oil, or other) by χ^2^ test for categorical variables or Kruskal-Wallis test for continuous variables to identify potential confounders. We also dichotomized semen parameters according to the lower reference limits of World Health Organization (WHO) 2010 reference levels^19^: volume less than 1.5 mL, concentration less than 15 million per mL, total sperm count less than 39 million, less than 32% progressively motile, and less than 4 morphologically normal spermatozoa, and we compared percentages of men below WHO reference limits according to supplement intake.

Data were analyzed using multiple linear regression analyses with each outcome separately. To meet model assumptions of normally distributed residuals and homoscedasticity, sperm concentration and total sperm count were transformed by cubic root, whereas all reproductive hormones were transformed by the natural logarithm. Covariates initially included factors possibly associated with semen parameters, reproductive hormone levels, or intake of fish oil supplements and were then excluded stepwise if they did not change the effect estimate by more than 10%. The same set of confounders was used for all semen parameters: duration of ejaculation abstinence (using linear splines for the slope: <48 hours, 48-96 hours, and >96 hours), age, fever within the last 3 months, self-rated physical fitness, and maternal and own smoking; for sperm motility, we also adjusted for duration between the time of ejaculation and analysis of the sample. Testis size was adjusted for age, fever in the past 3 months, BMI, and in utero maternal smoking exposure. The analyses of reproductive hormones were adjusted for time of blood sampling, smoking status, and BMI. Adding other factors did not change the estimates.

Owing to the cubic root transformations, the regression coefficients for sperm count are not easily interpretable and were back-transformed to show median total sperm count adjusted to a nonsmoking man without inutero maternal smoking exposure who was aged 19 years with self-reported very good or good physical fitness with no fever in the past 3 months and a mean abstinence period of 72 hours. As all reproductive hormones were transformed by use of the natural logarithm, the β coefficients were back-transformed to show the percentage differences among groups. Tests for trend among categories of men with no supplement use or use of fish oil supplements for fewer than 60 days or 60 or more days were performed excluding the other supplement category.

Finally, we stratified according to no intake, other supplements, multivitamins, fish oil supplements, or both fish oil and multivitamins to study effect modification and to test whether the combined intake would have an additive association with semen quality and reproductive hormones. Statistical analyses were performed using PASW GradPack statistical software version 22.0 (IBM Corp). The results are presented as regression coefficients with 95% CIs. *P* values were 2-sided, and *P* < .05 was considered statistically significant. We evaluated the fit of the regression models by testing the residuals for normality and by inspecting the residual plots. Data analysis was conducted from September 1, 2018, to June 30, 2019.

## Results

Thirty percent of men who were invited to participate agreed, and a total of 1694 men participated, of whom 1679 responded to the question about intake of vitamins or supplements. Median (IQR) age was 18.9 (18.7-19.4) years, median (IQR) BMI was 22.1 (20.3-24.0), and 871 men (52.0%) were daily or occasional smokers. There were 1125 men (67.0%) who did not use any supplements, 210 men (12.5%) who used multivitamins, 98 men (5.8%) who used fish oil supplements (of whom 37 [37.8%] reported intake on fewer than 60 days and 53 [54.1%] reported intake on 60 or more days), 70 men (4.2%) who used vitamin D supplements, and 25 men (1.5%) who used vitamin C supplements. A total of 306 men (18.2%) reported intake of supplements other than multivitamins or fish oil, while 150 men (8.9%) used only multivitamins, 38 men (2.3%) used only fish oil supplements, and 60 men (3.6%) used both fish oil supplements and multivitamins. Overall, there were no differences in semen quality or reproductive hormones among men who did not use dietary supplements compared with those who used vitamin C supplements, vitamin D supplements, or multivitamins (eTable in the Supplement). Compared with men with no supplement intake and men with other supplement intake, men who reported intake of fish oil supplements were generally older (median [IQR] age, 18.9 [18.7-19.3] years vs 19.0 [18.7-19.7] vs 19.0 [18.7-19.8] years; *P* = .12) and were less often daily smokers (319 men [28.4%] vs 78 men [17.1%] vs 11 men [11.2%]; *P* < .001) or exposed to smoking in utero (146 men [14.2%] vs 62 men [14.4%] vs 6 men [6.8%]; *P* = .15), although differences in age and in utero maternal smoking exposure were not significant. Self-report of health as good or very good was similar between men with fish oil supplement intake (86 men [87.8%]), men with no supplement intake (956 men [85.1%]), and men with other supplement intake (399 men [87.5%]) (*P* = .39). Men with fish oil supplement intake were more likely to self-report very good or good physical fitness (74 men [75.5%]) than men with no supplement intake (579 men [51.5%]) or men with other supplement intake (289 men [63.4%]) (*P* < .001). Men with fish oil supplement intake less often reported having had a fever within the last 3 months (6 men [6.2%]) than men with no supplement intake (83 men [7.4%]) or men with other supplement intake (62 men [13.7%]) (*P* = .001). There were no significant differences in self-report of having had a sexually transmitted disease, which was reported by 10 men with fish oil supplement intake (10.2%), 60 men with no supplement intake (5.3%), and 32 men with other supplement intake (32 men [7.0%]) (*P* = .16). Eighteen men with fish oil supplement intake (18.6%) had a BMI of 25 or higher, compared with 178 men with no supplement intake (16.1%) and 75 men with other supplement intake (16.9%) (*P* = .10), although the difference was not statistically significant (Table 1).

**Table 1.** Characteristics of Young Danish Men Stratified by Supplement Intake

Variable	No. (%)				*P* Value^a^
	All (N = 1679)	Supplement Intake			
		None (n = 1125)	Fish Oil (n = 98)	Other (n = 456)	
Information obtained at the physical examination					
Varicocele grade 2-3^b^	138 (8.4)	96 (8.7)	11 (11.3)	31 (7.0)	.30
Temperature >38 °C within past 3 mo	151 (9.0)	83 (7.4)	6 (6.2)	62 (13.7)	.001
BMI, median (IQR)	22.1 (20.3-24.0)	21.9 (20.2-23.9)	23.0 (21.1-24.6)	22.3 (20.6-24.1)	
<20	343 (20.8)	248 (22.4)	11 (11.3)	84 (18.9)	.10
20-24.99	1035 (62.8)	682 (61.6)	68 (70.1)	285 (64.2)	
≥25	271 (16.4)	178 (16.1)	18 (18.6)	75 (16.9)	
Information obtained from questionnaire					
Sexually transmitted disease^c^	102 (6.1)	60 (5.3)	10 (10.2)	32 (7.0)	.16
Born with cryptorchidism	37 (2.2)	22 (2.0)	2 (2.0)	13 (2.9)	.71
Age, median (IQR), y	18.9 (18.7-19.4)	18.9 (18.7-19.3)	19.0 (18.7-19.8)	19.0 (18.7-19.7)	.12
Alcohol intake, median (IQR), drinks/wk	6 (0-14)	6 (0-15)	4 (0-16)	5 (0-13)	.12
0	575 (34.2)	373 (33.2)	33 (33.7)	169 (37.1)	.37
1-14	699 (41.6)	467 (41.5)	40 (40.8)	192 (42.1)	
>14	405 (24.1)	285 (25.3)	25 (25.5)	95 (20.8)	
Self-rated health					
Very good or good	1441 (85.9)	956 (85.1)	86 (87.8)	399 (87.5)	.39
Average or poor	237 (14.1)	168 (14.9)	12 (12.2)	57 (12.5)	
Self-rated physical fitness					
Very good or good	942 (56.1)	579 (51.5)	74 (75.5)	289 (63.4)	<.001
Average	575 (34.3)	414 (36.8)	21 (21.4)	140 (30.7)	
Poor or very poor	161 (9.7)	131 (11.7)	3 (3.1)	27 (5.9)	
Smoking status					
None	804 (48.0)	506 (45.1)	56 (57.1)	242 (53.2)	<.001
Occasionally	463 (27.6)	297 (26.5)	31 (31.6)	135 (29.7)	
Daily	408 (24.4)	319 (28.4)	11 (11.2)	78 (17.1)	
Marijuana use					
None	1141 (68.2)	743 (66.3)	72 (73.5)	326 (71.6)	.06
Occasionally	489 (29.2)	341 (30.4)	25 (25.5)	123 (27.0)	
Daily	43 (2.6)	36 (3.2)	1 (1.0)	6 (1.3)	
Exposure to maternal smoking in utero	214 (13.8)	146 (14.2)	6 (6.8)	62 (14.4)	.15
Daily macronutrient intake, mean (SD)					
Total energy intake, kcal	2047 (856)	2029 (869)	2097 (748)	2082 (845)	.10
Energy from total fat, %	34.8 (5.9)	35.0 (6.0)	33.8 (5.3)	34.4 (5.9)	.03
Energy from protein, %	18.2 (3.3)	17.9 (3.0)	19.3 (3.5)	18.6 (3.6)	<.001
Energy from carbohydrates, %	46.2 (7.6)	46.7 (7.3)	45.7 (6.9)	46.1(8.2)	.75

Abbreviations: BMI, body mass index (calculated as weight in kilograms divided by height in meters squared); IQR, interquartile range.

^a^ Calculated using χ^2^ test for categorical variables or Kruskal-Wallis test for continuous variables.

^b^ Varicocele found at physical examination.

^c^ Sexually transmitted diseases included gonorrhea and chlamydia.

**Figure 1.**
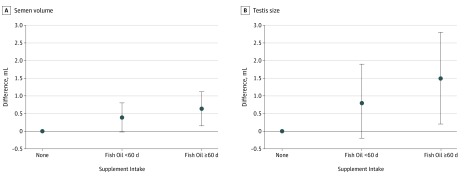
Adjusted Differences in Semen Volume and Testes Size Among Young Healthy Danish Men Stratified by Intake of Fish Oil Supplements During the Past 3 Months Multiple linear regression adjusted for period of abstinence, age, temperature greater than 38 °C in the past 3 months, self-rated physical fitness, and maternal and own smoking status. Men with no supplement intake were used as the reference group. Points indicate β; error bars, 95% CI.

**Figure 2.**
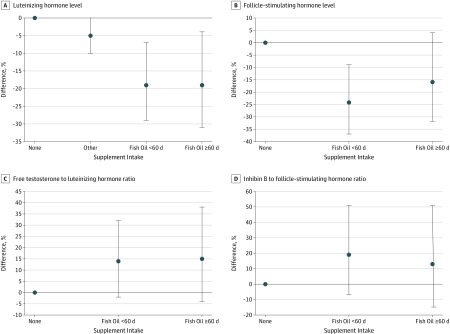
Adjusted Differences in Hormone Levels Among Young Healthy Danish Men Stratified by Intake of Fish Oil Supplements Multiple linear regression adjusted for time of blood sample delivery, body mass index, and smoking status. Hormone level measurements were transformed using the natural logarithm and back-transformed, giving percentage change. Men with no supplement intake were used as the reference group. Points indicate difference; error bars, 95% CI.

Men who reported intake of fish oil supplements had larger testes (β = 1.3 [95% CI, 0.5-2.1] mL), higher semen volume (β = 0.49 [95% CI, 0.18-0.80] mL), and higher cubic root transformed total sperm count (β = 0.39 [95% CI, 0.03-0.76] million) compared with men who did not use supplements (Table 2). Fewer men who used fish oil supplements had semen parameters below WHO reference limits: 2 men (2.0%) who reported fish oil supplement intake had semen volume less than 1.5 mL, compared with 84 men (7.5%) with no supplement intake, and 12 men (12.4%) who reported fish oil supplement intake had total sperm count less than 39 million, compared with 192 men (17.2%) with no supplement intake (eTable in the Supplement). In addition, men who used fish oil supplements had a 20% (95% CI, 9%-31%) lower FSH level, 16% (95% CI, 8%-24%) lower LH level, and 8% (95% CI, 0%-17%) higher free testosterone to LH ratio compared with men with no supplement intake (Table 3). No differences in inhibin B or testosterone levels were found (eTable in the Supplement). A dose-response association across fish oil supplement categories was present when we compared men with no supplement intake with men with fish oil supplement intake on fewer than 60 days vs 60 or more days, and men who reported intake of fish oil supplements for 60 or more days had the highest semen volume (<60 days: β = 0.38 [95% CI, −0.03 to 0.80] mL; ≥60 days: β = 0.64 [95% CI, 0.15-1.12] mL; *P* for trend <.001), highest cubic root transformed total sperm count (<60 days: β = 0.22 [95% CI, −0.27 to 0.72] million; ≥60 days: β = 0.76 [95% CI, 0.19-1.33] million; *P* for trend = .007), and largest testis size (<60 days: β = 0.8 [95% CI, −0.2 to 1.9] mL; ≥60 days: β = 1.5 [95% CI, 0.2-2.8] mL; *P* for trend = .007) (Table 2 and Figure 1). Using a man aged 19 years who did not smoke, was not exposed to smoking in utero, had self-reported good to very good physical fitness, no fever in the past 3 months, and a mean 72 hours of abstinence as the reference, total sperm count was 147 million for men with no supplement intake, 159 million for men with other supplement intake, 168 million for men with fish oil supplement intake on fewer than 60 days, and 184 million for men with fish oil supplement intake on 60 or more days. Compared with men with no supplement intake and after stratification according to intake of only multivitamins, only fish oil supplements, or both, men with intake of fish oil supplements only had the highest semen volume (only multivitamins: β = 0.23 [95% CI, −0.02 to 0.48] mL; only fish oil: β = 0.80 [95% CI, 0.33 to 1.27] mL; both: β = 0.27 [95% CI, −0.13 to 0.67] mL), cubic root transformed sperm concentration (only multivitamins: β = 0.02 [95% CI, −0.18 to 0.22] million/mL; only fish oil: β = 0.14 [95% CI, −0.24 to 0.51] million/mL; both: β = 0 [95% CI, −0.16 to 0.13] million/mL), cubic root transformed total sperm count (only multivitamins: β = 0.19 [95% CI, −0.10 to 0.49] million; only fish oil: β = 0.71 [95% CI, 0.16 to 1.27] million; both: β = 0.17 [95% CI, −0.30 to 0.64] million), and proportion of morphologically normal spermatozoa (only multivitamins: β = 0.7% [95% CI, −0.1% to 1.5%]; only fish oil: β = 1.0% [95% CI, −0.6% to 2.5%]; both: β = 0.3% [95% CI, −1.0% to 1.6%]) (Table 2). After adjustment, a dose-response association was found that suggested that men with more frequent intake of fish oil supplement had lower FSH (<60 days: −24% [95% CI, −37% to −9%]; ≥60 days: −16% [95% CI, −32% to 4%]) and LH (<60 days: −19% [95% CI, −29% to −7%]; ≥60 days: −19% [95% CI, −31% to −4%]) levels and a higher free testosterone to LH ratio (<60 days: 13% [95% CI, −2% to 31%]; ≥60 days: 15% [95% CI, −4% to 38%]) compared with men with no supplement intake (Table 2 and Figure 2).

**Table 2.** Adjusted Differences in Semen Quality and Testis Size Among Young Danish Men Stratified by Supplement Intake

Supplement Intake	β (95% CI)					
	Semen Volume, mL^a^	Sperm Concentration, million/mL^a^^,^^b^	Total Sperm Count, million^a^^,^^b^	Progressive Motile Spermatozoa, %^a^^,^^c^	Morphologically Normal Spermatozoa, %^a^	Testis Size, mL^d^
None	0 [Reference]	0 [Reference]	0 [Reference]	0 [Reference]	0 [Reference]	0 [Reference]
Fish oil	0.49 (0.18 to 0.80)	0.06 (−0.19 to 0.30)	0.39 (0.03 to 0.76)	−1.0 (−5.0 to 3.0)	0.6 (−0.4 to 1.6)	1.3 (0.5 to 2.1)
<60 d	0.38 (−0.03 to 0.80)	−0.04 (−0.38 to 0.29)	0.22 (−0.27 to 0.72)	−3.2 (−8.5 to 2.1)	0.6 (−0.7 to 2.0)	0.8 (−0.2 to 1.9)
≥60 d	0.64 (0.15 to 1.12)	0.27 (−0.11 to 0.66)	0.76 (0.19 to 1.33)	2.8 (−3.4 to 9.0)	0.7 (−0.8 to 2.3)	1.5 (0.2 to 2.8)
Other	0.21 (0.05 to 0.37)	0 (−0.13 to 0.12)	0.13 (−0.06 to 0.32)	−0.5 (−2.6 to 1.5)	0.1 (−0.4 to 0.6)	0.1 (−0.3 to 0.6)
*P* for trend^e^	<.001	.30	.007	.91	.21	.007
Combination vs single						
Multivitamins only	0.23 (−0.02 to 0.48)	0.02 (−0.18 to 0.22)	0.19 (−0.10 to 0.49)	−2.3 (−5.5 to 0.9)	0.7 (−0.1 to 1.5)	0.3 (−0.4 to 1.0)
Fish oil only	0.80 (0.33 to 1.27)	0.14 (−0.24 to 0.51)	0.71 (0.16 to 1.27)	−3.2 (−9.3 to 3.0)	1.0 (−0.6 to 2.5)	1.1 (−0.1 to 2.4)
Multivitamins and fish oil	0.27 (−0.13 to 0.67)	0 (−0.16 to 0.13)	0.17 (−0.30 to 0.64)	0.4 (−4.6 to 5.6)	0.3 (−1.0 to 1.6)	1.4 (0.3 to 2.4)
Other	0.20 (0.02 to 0.39)	−0.02 (−0.16 to 0.13)	0.10 (−0.12 to 0.32)	0.3 (−2.0:2.6)	−0.2 (−0.8 to 0.4)	0.1 (−0.4 to 0.6)

^a^ Multiple linear regression adjusted for period of abstinence, age, temperature greater than 38 °C within past 3 months, self-rated physical fitness, and maternal and own smoking status.

^b^ Transformed by the use of cubic root.

^c^ Additionally adjusted for duration between time of ejaculation and analysis of the sample.

^d^ Measured using ultrasonography and adjusted for age, temperature greater than 38 °C within past 3 months, maternal smoking, and body mass index.

^e^ Test for trend was performed by inserting the categorical fish oil supplement use variable into the model excluding the other supplement category, assuming the association to be linear.

**Table 3.** Adjusted Differences in Serum Reproductive Hormone Levels Among Young Danish Men Stratified by Supplement Intake

Supplement Intake	Difference, % (95% CI)						
	FSH^a^	LH^a^	FT^a^	Inhibin B^a^	FT to LH Ratio^a^	Inhibin B to FSH Ratio^a^	FT to Estradiol Ratio^a^
None	0 [Reference]	0 [Reference]	0 [Reference]	0 [Reference]	0 [Reference]	0 [Reference]	0 [Reference]
Fish oil	−20 (−31 to −9)	−16 (−24 to −8)	−1 (−7 to −6)	−5 (−13 to 3)	8 (0 to 17)	−2 (−14 to 12)	−7.5 (−14.2 to −0.4)
<60 d	−24 (−37 to −9)	−19 (−29 to −7)	1 (−8 to 10)	−8 (−17 to 3)	13 (−2 to 31)	19 (−7 to 51)	−7.8 (−16.4 to 1.7)
≥60 d	−16 (−32 to 4)	−19 (−31 to −4)	−3 (−13 to 9)	−2 (−14 to 11)	15 (−4 to 38)	13 (−15 to 51)	−7.3 (−17.8 to 4.4)
Other	−1 (−8 to 6)	−5 (−10 to −0)	2 (−2 to 5)	1 (−3 to 6)	8 (1 to 15)	9 (−2 to 21)	2.0 (−1.9 to 6.1)
*P* trend^b^	.003	<.001	.73	.25	.02	.15	.05
Combination vs single							
Multivitamins only	2 (−9 to 14)	−1 (−9 to 7)	5 (−1 to 11)	−6 (−12 to 1)	6 (−3 to 17)	−9 (−22 to 6)	−2.8 (−8.5 to 3.4)
Fish oil only	−14 (−30 to 6)	−12 (−25 to 3)	−6 (−15 to 5)	−3 (−14 to 10)	7 (−10 to 28)	13 (−15 to 50)	−8.3 (−18.4 to 2.8)
Multivitamins and fish oil	−24 (−36 to −10)	−19 (−29 to −8)	2 (−6 to 12)	−7 (−16 to 3)	14 (−2 to 31)	18 (−6 to 49)	−6.9 (−15.3 to 2.1)
Other	−3 (−11 to 6)	−7 (−13 to −1)	0 (−4 to 4)	5 (0 to 11)	8 (1 to 15)	8 (−3 to 21)	4.4 (−0.2 to 98.2)

Abbreviations: FSH, follicle-stimulating hormone; FT, free testosterone; LH, luteinizing hormone.

^a^ Multiple linear regression adjusted for smoking status, body mass index, and time of blood sample delivery. Transformed by use of the natural logarithm and back-transformed giving percentage change.

^b^ Test for trend was performed by inserting the categorical fish oil supplement use variable into the model excluding the other supplement category, assuming the association to be linear.

## Discussion

In this cross-sectional study, we found that intake of fish oil supplements during the past 3 months was associated with higher semen volume, total sperm count, and testis size; lower FSH and LH levels; and a higher free testosterone to LH ratio among young men compared with no supplement intake. Interestingly, a dose-response association was found, as men with intake of fish oil supplements on 60 or more days had higher semen volume, higher total sperm count, lower LH level, higher free testosterone level, and a lower free testosterone to LH ratio than men with fish oil intake on fewer than 60 days. Fewer men with fish oil intake had semen parameters below WHO reference levels than men with no supplement intake, and men with intake on 60 or more days had 0.6-mL-larger semen volume, 1.5-mL-larger testis, and a 15% higher free testosterone to LH ratio than men with no supplement intake, a difference that should be considered of clinical relevance.

Men with intake of other supplements (ie, vitamin C, vitamin D, or multivitamins) did not have significantly different semen quality or reproductive hormone profiles than men with no supplement intake. This suggests that our findings are unlikely to be explained by confounding by indication, whereby men with intake of supplements have a healthier lifestyle and better health behavior, factors that may also affect their semen quality and reproductive hormones. This was indeed the case, as men who reported intake of supplements were less likely to smoke and were less exposed to smoking in utero. They had better self-reported health and physical fitness and higher BMI. However, we adjusted for these variables. Nevertheless, we cannot exclude confounding by unmeasured lifestyle or behavioral factors.

The association of intake of fish oil supplements with lower FSH levels but not with a difference in inhibin B levels indicates a potential benefit of ω-3 fatty acid on spermatogenic capacity and testicular function. This finding is compatible with our finding of higher sperm count among men with fish oil supplement intake. Thus, fish oil supplement use may be associated with increased FSH sensitivity of the Sertoli cells. Similarly, the lower LH level and higher free testosterone to LH ratio indicate a better Leydig cell capacity in line with a positive association with fish oil supplement intake.

Our findings are in line with previous studies. Among men presenting in fertility clinics, fish intake was positively associated with total sperm count and higher proportion of morphologically normal spermatozoa,^23^ and higher intake of ω-3 PUFAs was associated with a higher proportion of morphologically normal sperm cells.^24^ One RCT^11^ found that long-chain ω-3 fatty acids (DHA and eicosapentaenoic acid) supplementation among men with idiopathic oligoasthenoteratospermia resulted in a significant increase in total sperm count, sperm concentration, and percentages of motile and morphologically normal spermatozoa. In 2 RCTs among men with infertility,^12,13^ DHA supplementation decreased sperm DNA fragmentation index and increased progressively motile sperm cells. However, in a small RCT among 28 men with asthenospermia, 3 months of DHA supplementation did not improve sperm motility.^25^ In 2 RCTs of young healthy men consuming a typical Western-style diet, participants were randomized to intake of 75 g/day of walnuts, which are rich in α-linolenic acids, or 60 g/day of nut mix (ie, walnuts, almonds, and hazelnuts) for approximately 3 months.^14,15^ Both studies^14,15^ found improvements in sperm vitality, motility, and percentage of morphologically normal spermatozoa, and 1 study^15^ found an increase in total sperm count and decrease in DNA fragmentation in nut intake groups compared with the control groups. In addition, high intake of saturated fat has been associated with lower total sperm count and concentration among US patients with infertility^24^ and healthy young men in Denmark.^26^

The rich fatty acid content of the sperm cell membrane is critical for sperm function.^27^ The sperm cell membrane plays a critical role in key fertilization events, such as capacitation, acrosome reaction, and sperm-oocyte fusion.^28^ The content of PUFAs, particularly of DHA, in the sperm membrane increases as the sperm matures.^29^ These changes are due to local metabolism as well as dietary input.^30^ However, PUFAs cannot be synthesized endogenously in humans; therefore, they must be obtained from other sources, including consuming seafood for longer chain ω-3 PUFAs, such as eicosapentaenoic acid and DHA, or consuming seeds, nuts, and vegetable oils in the case of 18-carbon linoleic acid and α-linolenic acids. Diets rich in α-linolenic acids or fish oil, which is rich in eicosapentaenoic acid and DHA, have been found to increase testicular DHA in rodent models^30^ as well as sperm membrane DHA in humans.^11^ Higher DHA in the sperm membrane has been associated with increased sperm motility, morphologically normal spermatozoa, and sperm concentration.^11,31,32,33,34,35,36^ In addition, a 2018 study found a positive correlation of cholesterol levels in seminal plasma and sperm count.^37^

### Strengths and Limitations

Our study had some strengths, including that it was large and included young, unselected, and healthy men. Our participation rate was similar to other population-based studies of semen quality. In addition, most of the young men included in the study had no knowledge of their own fertility potential, so this is unlikely to have affected their motivation to participate.

However, our study also had some limitations. Few men reported intake of fish oil without multivitamins, and we had no information on the actual concentration of ω-3 fatty acids in the fish oil supplement, which may have introduced misclassification, as supplements may contain different concentrations of ω-3 fatty acids. However, this likely resulted in randomly misclassifying some individuals with low intake as high intake and vice versa, thereby resulting in underestimation of the association of fish oil with reproductive health and testicular function. Fish oil supplements (eg, cod liver oil) may also contain vitamin A, which has been associated with semen quality.^10^ However, in Denmark most fish oil supplements are sold as capsules that include only long-chained ω-3 PUFAs without other ingredients; therefore, we do not believe that this is a likely explanation for our findings. Also, no association with intake of multivitamins, which contain more vitamin A than fish oil supplements do, was found. In addition, we did not adjust for dietary fish intake, as this was not available for all men. Nevertheless, fish intake among these young men and the general population in Denmark is generally low (mean, 26 grams of fish per day) (F. L. Nassan, PhD, unpublished data, 2019).

It is well known that interobserver variability in semen analysis exists and is particularly high for morphologic and motility assessment, which may help explain the lack of an association of fish oil intake with these semen measures. However, all analyses were blinded to men’s exposures, and the same 2 trained technicians assessed all morphologic slides. We obtained only 1 semen sample from each man. However, intraindividual variability of semen would increase the variation, thereby introducing a bias toward the null hypothesis owing to potential nondifferential misclassification. Also, there is a known circadian rhythm in hormone production; however, blood samples were mostly taken in the mornings, and we adjusted for sampling times in the analysis. The process of spermatogenesis takes approximately 72 days, followed by a further maturation in the epididymis; therefore, we believe that the questionnaire addressing intake of supplements during 3 months before delivering the sample is a good marker of the exposure window.

## Conclusions

In this large cross-sectional study, we found positive associations of self-reported use of fish oil supplements with testicular function as measured by higher semen volume, total sperm count, and testis size, lower FSH and LH levels, and a higher free testosterone to LH ratio. As we found no clear associations of intake of other supplements with measures of semen quality, we believe that confounding by indication is not likely to explain our findings. However, we did not obtain information about the actual content of ω-3 fatty acids in the supplements, and the study was cross-sectional, so we can only claim associations and not causation. To our knowledge, RCTs on intake of fish oil supplements have only been performed among men with infertility, and our findings need to be confirmed in well-designed RCTs among unselected men or in large prospective cohort studies.

## Notes

Supplement. **eTable.** Semen Quality, Testis Size, and Reproductive Hormone Levels Among Young Danish Men Stratified by Intake of Multivitamin, Vitamin C, or Vitamin D Supplements

## References

[1] Légaré C, Droit A, Fournier F, Investigation of male infertility using quantitative comparative proteomics. J Proteome Res. 2014;13(12):-. doi:10.1021/pr501031x 25355644

[2] Thonneau P, Marchand S, Tallec A, Incidence and main causes of infertility in a resident population (1,850,000) of three French regions (1988-1989). Hum Reprod. 1991;6(6):811-816. doi:10.1093/oxfordjournals.humrep.a137433 1757519

[3] Carlsen E, Giwercman A, Keiding N, Skakkebaek NE Evidence for decreasing quality of semen during past 50 years. BMJ. 1992;305(6854):609-613. doi:10.1136/bmj.305.6854.609 1393072 PMC1883354

[4] Levine H, Jørgensen N, Martino-Andrade A, Temporal trends in sperm count: a systematic review and meta-regression analysis. Hum Reprod Update. 2017;23(6):646-659. doi:10.1093/humupd/dmx022 28981654 PMC6455044

[5] Hayden RP, Flannigan R, Schlegel PN The role of lifestyle in male infertility: diet, physical activity, and body habitus. Curr Urol Rep. 2018;19(7):56. doi:10.1007/s11934-018-0805-0 29774489

[6] Ricci E, Al Beitawi S, Cipriani S, Semen quality and alcohol intake: a systematic review and meta-analysis. Reprod Biomed Online. 2017;34(1):38-47. doi:10.1016/j.rbmo.2016.09.012 28029592

[7] Bundhun PK, Janoo G, Bhurtu A, Tobacco smoking and semen quality in infertile males: a systematic review and meta-analysis. BMC Public Health. 2019;19(1):36. doi:10.1186/s12889-018-6319-3 30621647 PMC6325781

[8] Salas-Huetos A, Bulló M, Salas-Salvadó J Dietary patterns, foods and nutrients in male fertility parameters and fecundability: a systematic review of observational studies. Hum Reprod Update. 2017;23(4):371-389. doi:10.1093/humupd/dmx006 28333357

[9] Gaskins AJ, Chavarro JE Diet and fertility: a review. Am J Obstet Gynecol. 2018;218(4):379-389. doi:10.1016/j.ajog.2017.08.010 28844822 PMC5826784

[10] Hogarth CA, Griswold MD The key role of vitamin A in spermatogenesis. J Clin Invest. 2010;120(4):956-962. doi:10.1172/JCI41303 20364093 PMC2846058

[11] Safarinejad MR Effect of omega-3 polyunsaturated fatty acid supplementation on semen profile and enzymatic anti-oxidant capacity of seminal plasma in infertile men with idiopathic oligoasthenoteratospermia: a double-blind, placebo-controlled, randomised study. Andrologia. 2011;43(1):38-47. doi:10.1111/j.1439-0272.2009.01013.x 21219381

[12] Martínez-Soto JC, Domingo JC, Cordobilla B, Dietary supplementation with docosahexaenoic acid (DHA) improves seminal antioxidant status and decreases sperm DNA fragmentation. Syst Biol Reprod Med. 2016;62(6):387-395. doi:10.1080/19396368.2016.1246623 27792396

[13] González-Ravina C, Aguirre-Lipperheide M, Pinto F, Effect of dietary supplementation with a highly pure and concentrated docosahexaenoic acid (DHA) supplement on human sperm function. Reprod Biol. 2018;18(3):282-288. doi:10.1016/j.repbio.2018.06.002 29934046

[14] Robbins WA, Xun L, FitzGerald LZ, Esguerra S, Henning SM, Carpenter CL Walnuts improve semen quality in men consuming a Western-style diet: randomized control dietary intervention trial. Biol Reprod. 2012;87(4):101. doi:10.1095/biolreprod.112.101634 22895856

[15] Salas-Huetos A, Moraleda R, Giardina S, Effect of nut consumption on semen quality and functionality in healthy men consuming a Western-style diet: a randomized controlled trial. Am J Clin Nutr. 2018;108(5):953-962. doi:10.1093/ajcn/nqy181 30475967

[16] World Medical Association World Medical Association Declaration of Helsinki: ethical principles for medical research involving human subjects. JAMA. 2013;310(20):2191-2194. doi:10.1001/jama.2013.281053. 24141714

[17] Priskorn L, Nordkap L, Bang AK, Average sperm count remains unchanged despite reduction in maternal smoking: results from a large cross-sectional study with annual investigations over 21 years. Hum Reprod. 2018;33(6):998-1008. doi:10.1093/humrep/dey090 29659832

[18] Jensen TK, Gottschau M, Madsen JO, Habitual alcohol consumption associated with reduced semen quality and changes in reproductive hormones: a cross-sectional study among 1221 young Danish men. BMJ Open. 2014;4(9):e005462. doi:10.1136/bmjopen-2014-005462 25277121 PMC4185337

[19] World Health Organization WHO Laboratory Manual for the Examination and Processing of Human Semen. 5th ed Geneva, Switzerland: World Health Organization Department of Reproductive Health and Research; 2010.

[20] Jørgensen N, Joensen UN, Jensen TK, Human semen quality in the new millennium: a prospective cross-sectional population-based study of 4867 men. BMJ Open. 2012;2(4):e000990. doi:10.1136/bmjopen-2012-000990 22761286 PMC3391374

[21] Menkveld R, Swanson RJ, Kotze TJ, Kruger TF Comparison of a discontinuous Percoll gradient method versus a swim-up method: effects on sperm morphology and other semen parameters. Andrologia. 1990;22(2):152-158. doi:10.1111/j.1439-0272.1990.tb01957.x 2176068

[22] Vermeulen A, Verdonck L, Kaufman JM A critical evaluation of simple methods for the estimation of free testosterone in serum. J Clin Endocrinol Metab. 1999;84(10):3666-3672. doi:10.1210/jcem.84.10.6079 10523012

[23] Afeiche MC, Gaskins AJ, Williams PL, Processed meat intake is unfavorably and fish intake favorably associated with semen quality indicators among men attending a fertility clinic. J Nutr. 2014;144(7):1091-1098. doi:10.3945/jn.113.190173 24850626 PMC4056648

[24] Attaman JA, Toth TL, Furtado J, Campos H, Hauser R, Chavarro JE Dietary fat and semen quality among men attending a fertility clinic. Hum Reprod. 2012;27(5):1466-1474. doi:10.1093/humrep/des065 22416013 PMC3329193

[25] Conquer JA, Martin JB, Tummon I, Watson L, Tekpetey F Effect of DHA supplementation on DHA status and sperm motility in asthenozoospermic males. Lipids. 2000;35(2):149-154. doi:10.1007/BF02664764 10757545

[26] Jensen TK, Heitmann BL, Blomberg Jensen M, High dietary intake of saturated fat is associated with reduced semen quality among 701 young Danish men from the general population. Am J Clin Nutr. 2013;97(2):411-418. doi:10.3945/ajcn.112.042432 23269819

[27] Nassan FL, Chavarro JE, Tanrikut C Diet and men’s fertility: does diet affect sperm quality? Fertil Steril. 2018;110(4):570-577. doi:10.1016/j.fertnstert.2018.05.025 30196939

[28] Flesch FM, Gadella BM Dynamics of the mammalian sperm plasma membrane in the process of fertilization. Biochim Biophys Acta. 2000;1469(3):197-235. doi:10.1016/S0304-4157(00)00018-6 11063883

[29] Lenzi A, Gandini L, Maresca V, Fatty acid composition of spermatozoa and immature germ cells. Mol Hum Reprod. 2000;6(3):226-231. doi:10.1093/molehr/6.3.226 10694269

[30] Sebokova E, Garg ML, Wierzbicki A, Thomson AB, Clandinin MT Alteration of the lipid composition of rat testicular plasma membranes by dietary (n-3) fatty acids changes the responsiveness of Leydig cells and testosterone synthesis. J Nutr. 1990;120(6):610-618. doi:10.1093/jn/120.6.610 2352035

[31] Conquer JA, Martin JB, Tummon I, Watson L, Tekpetey F Fatty acid analysis of blood serum, seminal plasma, and spermatozoa of normozoospermic vs. asthenozoospermic males. Lipids. 1999;34(8):793-799. doi:10.1007/s11745-999-0425-1 10529089

[32] Zalata AA, Christophe AB, Depuydt CE, Schoonjans F, Comhaire FH The fatty acid composition of phospholipids of spermatozoa from infertile patients. Mol Hum Reprod. 1998;4(2):111-118. doi:10.1093/molehr/4.2.111 9542967

[33] Gulaya NM, Margitich VM, Govseeva NM, Klimashevsky VM, Gorpynchenko II, Boyko MI Phospholipid composition of human sperm and seminal plasma in relation to sperm fertility. Arch Androl. 2001;46(3):169-175. doi:10.1080/01485010151096405 11339641

[34] Tavilani H, Doosti M, Nourmohammadi I, Lipid composition of spermatozoa in normozoospermic and asthenozoospermic males. Prostaglandins Leukot Essent Fatty Acids. 2007;77(1):45-50. doi:10.1016/j.plefa.2007.07.001 17693070

[35] Tavilani H, Doosti M, Abdi K, Vaisiraygani A, Joshaghani HR Decreased polyunsaturated and increased saturated fatty acid concentration in spermatozoa from asthenozoospermic males as compared with normozoospermic males. Andrologia. 2006;38(5):173-178. doi:10.1111/j.1439-0272.2006.00735.x 16961570

[36] Aksoy Y, Aksoy H, Altinkaynak K, Aydin HR, Ozkan A Sperm fatty acid composition in subfertile men. Prostaglandins Leukot Essent Fatty Acids. 2006;75(2):75-79. doi:10.1016/j.plefa.2006.06.002 16893631

[37] de Neergaard R, Nielsen JE, Jørgensen A, Toft BG, Goetze JP, Jørgensen N Positive association between cholesterol in human seminal plasma and sperm counts: results from a cross-sectional cohort study and immunohistochemical investigations. Andrology. 2018;6(6):817-828. doi:10.1111/andr.12532 30182437

