# Supplementary material for: Associations of Fish Oil Supplement Use With Testicular Function in Young Men
Source: JAMA Netw Open. 2020 Jan 17;3(1):e1919462. doi: 10.1001/jamanetworkopen.2019.19462 (PMC6991322; doi:10.1001/jamanetworkopen.2019.19462)
Supplement: Supplement. — eTable. Semen Quality, Testis Size, and Reproductive Hormone Levels Among Young Danish Men Stratified by Intake of Multivitamin, Vitamin C, or Vitamin D Supplements [file jamanetwopen-3-e1919462-s001.pdf]

Supplementary Online Content

Jensen TK, Priskorn L, Holmboe SA, et al. Associations of fish oil supplement use with testicular function in young men. *JAMA Netw Open*. 2020;3(1):e1919462. doi:10.1001/jamanetworkopen.2019.19462

eTable. Semen Quality, Testis Size, and Reproductive Hormone Levels Among Young Danish Men Stratified by Intake of Multivitamin, Vitamin C, or Vitamin D Supplements

This supplementary material has been provided by the authors to give readers additional information about their work.

© 2020 Jensen TK et al. *JAMA Network Open.*

eTable. Semen Quality, Testis Size, and Reproductive Hormone Levels Among Young Danish Men Stratified by Intake of Multivitamin, Vitamin C, or Vitamin D Supplements

Variables	All N=1679	Intake of supplements				
		None N=1125	Fish oil N=98	Multivitamin N=210	Vitamin C N=25	Vitamin D N=70
**Semen quality:**						
Testis size (ml) US[1]	13 (11;16)	13 (11;16)	14 (12;17)	14 (11;16)	13 (11;16)	14 (12;16)
Semen volume (ml)	3.2 (2.3;4.2)	3.1 (2.3;4.0)	3.6 (3.0;4.7)	3.5 (2.6;4.3)	3.4 (3.0;4.8)	3.4 (2.2;4.2)
Sperm concentration (mill/ml)	42 (20;73)	42 (20;71)	46 (20;85)	43 (20;87)	45 (15;65)	38 (20;72)
Total sperm count (mill)	129 (59;233)	124 (56;224)	166 (74;308)	151 (64;287)	108 (43;233)	127 (56;239)
Progressive motile sperm (%)	62.7 (50.0;71.3)	62.7 (50.3;71.3)	63.2 (50.2;71.8)	60.0 (48.7;70.0)	63.7 (48.5;77.0)	60.3 (47.7;71.5)
Morphologically normal forms (%)	6.5 (3.5;9.5)	6.5 (3.5;9.5)	7.5 (4.1;9.5)	6.8 (3.5;11.0)	6.5 (2.5;10.8)	6.0 (3.9;9.0)
Period of abstinence (hours)	61 (57;82)	61 (56;82)	61 (58;84)	61 (58;84)	65 (59;86)	60 (55;78)
Duration between time of ejaculation and analysis of the sample (% > 1 hour)	7.7	6.8	18.5	11.3	8.0	8.7
**N (%) below WHO reference levels**						
Semen volume<1.5 ml	109 (6.5)	84 (7.5)	2 (2.0)	10 (4.8)	1 (4.0)	8 (11.4)
Sperm concentration<15 mill/ml	313 (18.7)	212 (18.9)	15 (15.5)	39 (18.7)	6 (24.0)	11 (15.9)
Total sperm count <39 mill	211 (16.7)	192 (17.2)	12 (12.4)	33 (15.8)	5 (20.0)	10 (14.5)
Percent progressive motile<32	139 (8.4)	88 (7.9)	10 (10.2)	16 (7.7)	3 (12.0)	8 (11.4)
Percent morphological normal<4	435 (26.3)	286 (25.9)	22 (22.9)	52 (25.2)	9 (36.0)	17 (24.3)
**Reproductive hormones**						
FSH (IU/L)	2.7 (1.8;3.8)	2.7 (1.8;3.8)	2.4 (1.7;3.3)	2.7 (1.8;4.0)	2.8 (2.0;3.6)	2.7 (1.8;3.8)
LH (IU/L)	3.3 (2.5;4.5)	3.4 (2.5;4.6)	3.1 (2.1;4.3)	3.3 (2.5;4.3)	2.8 (2.1;4.0)	3.2 (2.3;4.2)
Testosterone (nmol/L)	20.9 (17.1;24.6)	21.1 (12.6;32.2)	20.6 (16.9;24.1)	20.6 (17.2;25.1)	20.0 (16.8;24.4)	20.6 (16.3;24.1)
SHBG (nmol/L)	34 (26;43)	34 (26;43)	33 (26;42)	32 (26;41)	36 (30;48)	34 (26;41)
Free testosterone (pmol/L)	437 (361;519)	441 (362;523)	434 (365;514)	447 (363;526)	409 (360;475)	431 (370;499)
Inhibin B (pg/mL)	170 (82;281)	170 (134;213)	157 (133;198)	159 (131;204)	175 (126;201)	172 (126;210)
Estradiol (nmol/L)	83 (64;102)	83 (64;102)	87 (71;109)	85 (70;106)	88 (66;102)	87 (69;105)
Free testosterone/LH	132 (96;178)	131 (94;176)	146 (105;197)	135 (98;184)	149 (102;198)	139 (98;193)
Inhibin B/FSH	65 (37;109)	63 (37;109)	66 (40;114)	61 (34;107)	58 (29;97)	58 (39;100)
Free testosterone/estradiol	5.3 (4.3;6.7)	5.3 (4.4;6.7)	5.1 (4.1;6.3)	5.2 (4.1;6.2)	5.1 (4.0;6.0)	5.0 (4.1;6.0)

© 2020 Jensen TK et al. *JAMA Network Open.*

Time of blood sampling	10:20 (9:40;11:10)	9:50 (9:15;10:35)	10:41 (10:05;11:20)	10:00 (9:20;10:40)	10:33 (9:55;11:25)	10:00 (9:35;11:05)

[1]Mean of the two testes.
Abbreviation, N; number of men, US; Ultrasound.

© 2020 Jensen TK et al. *JAMA Network Open.*
